# Reduced pulmonary vascular reserve during stress echocardiography in confirmed pulmonary hypertension and patients at risk of overt pulmonary hypertension

**DOI:** 10.1007/s10554-020-01897-3

**Published:** 2020-05-27

**Authors:** Karina Wierzbowska-Drabik, Jarosław D. Kasprzak, Michele D′Alto, Gergely Ágoston, Albert Varga, Francesco Ferrara, Miguel Amor, Quirino Ciampi, Eduardo Bossone, Eugenio Picano

**Affiliations:** 1 I Department and Chair of Cardiology, Medical University of Lodz, Bieganski Hospital, Lodz, Poland; 2 Department of Cardiology, University “L. Vanvitelli”- AORN dei Colli - Monaldi Hospital, Naples, Italy; 3 Department of Family Medicine, University of Szeged, Tisza Lajos krt. 109, Szeged, 6725 Hungary; 4 Cardiology Division, Heart Department, University Hospital of Salerno, “Cava de′ Tirreni and Amalfi Coast” Hospital, Salerno, Italy; 5 Cardiology Department, Ramos Mejia Hospital, Buenos Aires, Argentina; 6 Division of Cardiology, Fatebenefratelli Hospital, Benevento, Italy; 7 AORN A. Cardarelli Hospital, Naples, Italy; 8 Institute of Clinical Physiology - C.N.R., Pisa, Italy

**Keywords:** Pulmonary hypertension, Stress echocardiography, Pulmonary vascular reserve, Tricuspid regurgitant velocity, Pulmonary acceleration time

## Abstract

Noninvasive estimation of systolic pulmonary artery pressure (SPAP) during exercise stress echocardiography (ESE) is recommended for pulmonary hemodynamics evaluation but remains flow-dependent. Our aim was to assess the feasibility of pulmonary vascular reserve index (PVRI) estimation during ESE combining SPAP with cardiac output (CO) or exercise-time and compare its value in three group of patients: with invasively confirmed pulmonary hypertension (PH), at risk of PH development (PH risk) mainly with systemic sclerosis and in controls (C) without clinical risk factors for PH, age-matched with PH risk patients. We performed semisupine ESE in 171 subjects: 31 PH, 61 PH at risk and 50 controls as well as in 29 young, healthy normals. Rest and stress assessment included: tricuspid regurgitant flow velocity (TRV), pulmonary acceleration time (ACT), CO (Doppler-estimated). SPAP was calculated from TRV or ACT when TRV was not available. We estimated PVRI based on CO (peak CO/SPAP*0.1) or exercise-time (ESE time/SPAP*0.1). During stress, TRV was measurable in 44% patients ACT in 77%, either one in 95%. PVRI was feasible in 65% subjects with CO and 95% with exercise-time (p < 0.0001). PVRI was lower in PH compared to controls both for CO-based PVRI (group 1 = 1.0 ± 0.95 vs group 3 = 4.28 ± 2.3, p < 0.0001) or time-based PVRI estimation (0.66 ± 0.39 vs 3.95 ± 2.26, p < 0.0001). The proposed criteria for PH detection were for CO-based PVRI ≤ 1.29 and ESE-time based PVRI ≤ 1.0 and for PH risk ≤ 1.9 and ≤ 1.7 respectively. Noninvasive estimation of PVRI can be obtained in near all patients during ESE, without contrast administration, integrating TRV with ACT for SPAP assessment and using exercise time as a proxy of CO. These indices allow for comparison of pulmonary vascular dynamics in patients with varied exercise tolerance and clinical status.

## Introduction

The disproportionate increase in pulmonary artery pressure during exercise is probably a precursor to pulmonary hypertension (PH) at rest [1, 2]. The early detection of PH is especially important in patients belonging to group 1 by the updated clinical classification of PH, including relatives of patients with diagnosed PAH (pulmonary arterial hypertension) as well as PH associated to other diseases such as systemic sclerosis [1–4]. These patients presents high risk of PH development, assessed for 8% to 12%, and earlier therapy may improve their outcomes [5].

Nevertheless, noninvasive, echocardiographic assessment of systolic arterial pulmonary pressure (SPAP) has the intrinsic limitation of being flow-dependent and in such a way by definition workload- related [1]. In spite of the many advantages as a noninvasive, available, radiation-free technique, the use of exercise stress echocardiography (ESE) has remained limited to date, mostly for technical problems of sampling tricuspid regurgitant velocity (TRV) during exercise [6] and the conceptual limitation of estimating pulmonary hemodynamics with a flow-dependent index such as SPAP, loosing moreover its close relationship with pulmonary pressure in most advanced tricuspid regurgitation [1–7]. The feasibility problem of TRV may, however, be minimized by measuring the acceleration time (ACT) of physiologic forward systolic pulmonary flow velocity, also present when TRV is unfeasible and estimating SPAP with a combined TRACT (tricuspid regurgitation + acceleration time) approach [8, 9].

The feature of pathologic increase of SPAP during exercise is its disproportion to the cardiac output (CO) augmentation and abnormal response of pulmonary vascular resistance. It has been estimated that normal pulmonary vascular response is related with the increase of mean pulmonary arterial pressure in the range of 1–2 mmHg per 1 l/min augmentation of CO [10]. Recently, the noninvasive assessment of similar parameter was proposed since the CO can be estimated from 2D volumetric echocardiography or preferably by Doppler, or even through the proxy of minutes of exercise [11].

The hypothesis of the present study is that in its refined version, ESE can solve the two major problems of suboptimal success rate for SPAP estimation as well as the limited discriminatory capacity of flow-dependent parameters.

The primary aim of our study was to assess in a multicenter, prospective design the feasibility of pulmonary vascular reserve index (PVRI) based either on CO (CO/SPAP*0.1 at peak stress) or on simpler, imaging-independent exercise time (ESE time/SPAP*0.1 at peak stress). The secondary aim was to evaluate the value of these parameters in differentiating pulmonary hemodynamic patterns in patients with either confirmed PH or at risk for PH development compared to an age-matched control group.

## Methods

The study was performed as the part of the multicentre project [12, 13] and its protocol was reviewed and approved by the institutional ethics committees as a part of the SE 2020 study (148-Comitato Etico Lazio-1, July 16, 2016; Clinical trials.Gov Identifier NCT 030.49995). All patients gave informed consent to enter the study.

### Study group

We included 142 subjects, 96 women, mean age 55 ± 15 years (from 19 to 88) from six centers, without the contraindications to exercise referred to ESE for the assessment of exercise tolerance as well as for CAD diagnosis/exclusion. As PH group we examined 31 consecutive patients with diagnosed, treated and monitored PH as confirmed with mPAP > 25 mmHg assessed by right heart catheterization whose were amenable to exercise tolerance assessment with ESE.

Into PH risk group we included 61 subjects with systemic sclerosis (56 subjects) or mixed connective tissue disease (five patients) and as a control group 50 age-matched subjects without risk factors of PH but with present risk factors for cardiovascular disease although without diagnosed coronary artery disease. Additionally we evaluated also the group of 29 young, healthy subjects, without risk factors for cardiovascular diseases, whose data were used for establishing the normal values of tested parameters (PVRI CO and ESE time based).

### Echocardiographic assessment

Transthoracic echocardiography at rest and ESE was performed with E9 (GE, Vingmed, Norway) or VIVID 7 (GE Vingmed Ultrasound AS, Horten, Norway) systems using M4S/M5S probes. During the echocardiography an ECG tracing was displayed on the monitor. The echocardiographic measurements (including Doppler parameters) were taken following the recommendations [6, 14–16].

### Assessment of TRV and ACT

TRV was derived with continuous wave Doppler from the apical four chamber or the parasternal right ventricular inflow view. The right ventricular systolic pressure was assumed to equal SPAP, and was calculated with the Bernoulli equation (TRV in m/s): SPAP = 4 TRV^2^ + right atrial pressure (RAP). RAP was estimated from the inferior vena cava (IVC) diameter and collapsing during the inspiration. We added RAP of 3 mm Hg (IVC diameter < 2.1 cm, collapsing > 50% with a sniff), or 15 mm Hg (diameter > 2.1 cm, collapsing ≤ 50% with a sniff) or 8 mm Hg in mixed scenarios. The value calculated at rest was applied also at stress [6, 17].

ACT was measured in right ventricular outflow tract as the time (in milliseconds) from the beginning of pulmonary ejection until the peak value of systolic velocity. Pulsed wave Doppler was used with sample positioned at the annulus of the pulmonary artery, in the parasternal short axis or in the subcostal view. The normal value is > 110 ms, the abnormal < 105, and indeterminate—between 105 and 110 ms [18]. From the raw data of ACT, SPAP was derived on the basis of the correlation linking ACT to TRV as follows: log_10_ SPAP = − 0.004 (ACT) + 2.1 [19].

### Exercise stress echocardiography

**Fig. 1:**
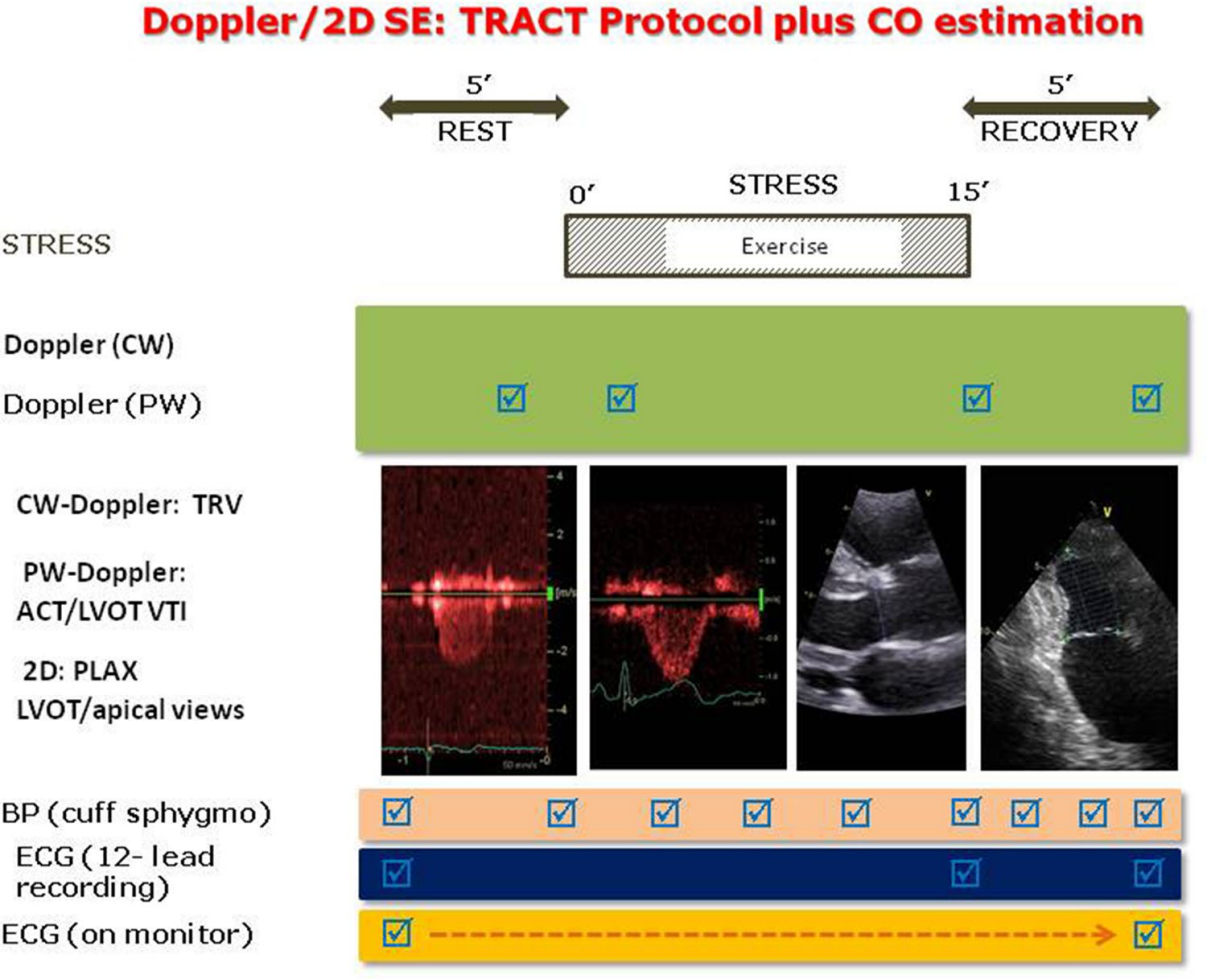
The study protocol with assessment of TRV and ACT for SPAP estimation, and cardiac output with Doppler/2D method. Exercise time is also recorded. *ACT* acceleration time, *BP* blood pressure, *CO* cardiac output, *CW* continuous wave, *ECG* electrocardiogram, *LVOT* left ventricular outflow tract, *PLAX* parasternal long axis view, *PW* pulsed wave, *SE* stress echocardiography, *TRACT* acronym from tricuspid regurgitation/acceleration time, *TRV* tricuspid regurgitant velocity, *VTI* velocity time integral

All patients underwent semi-supine bicycle ESE as described by recent recommendations [14]. The study protocol combining TRV and ACT assessment (TRACT) is displayed in Fig. 1. ESE was performed at an initial workload of 25 Watts lasting for 2 min, then the workload increased stepwise by 25 Watts at 2-min intervals. Electrocardiogram and blood pressure were monitored and all studies were performed by cardiologists experienced in different kind of stress echo examinations and analysis assisted by the nurses or the doctors. Criteria for interrupting the test were chest pain, induced wall motion abnormalities, significant rhythm disturbances, excessive fatigue, blood pressure increase (systolic ≥ 240 mmHg, diastolic ≥ 120 mmHg), limiting dyspnea, legs pain or predicted heart rate. Video loops of heart cycles were acquired and digitally stored for further analysis. All echocardiographers had passed quality control of reading examinations as required by Stress Echo 2020 study with interobserver reproducibility ≥ 90% [20]. CO was derived from the Doppler-estimated stroke volume (SV) using the velocity time integral of flow through the left ventricular outflow tract [21–25]. Pulmonary vascular resistance (PVR) was calculated using Abbas formula: *PVR (Wood Units)* = *TRV *(m/s)*/VTI RVOT *(cm) *10 + 0.16 and assessed separately in two time points at baseline and peak exercise [26, 27].

As relatively flow-independent SPAP derived parameter we modified and simplified those proposed by Claessens: SPAP/CO slope (requiring two points, rest and peak, and slope calculation) was modified into CO/peak SPAP*0.1; SPAP/exercise intensity was modified into ESE time/peak SPAP*0.1 [11]. Pulmonary vascular reserve index (PVRI) was estimated as CO-based (CO-PVRI) or as exercise-time-based (ESE time-PVRI). ESE-time was defined as time in minutes from beginning to the peak of exercise. Higher values of these parameters may reflect better pulmonary vascular reserve.

**Fig. 2:**
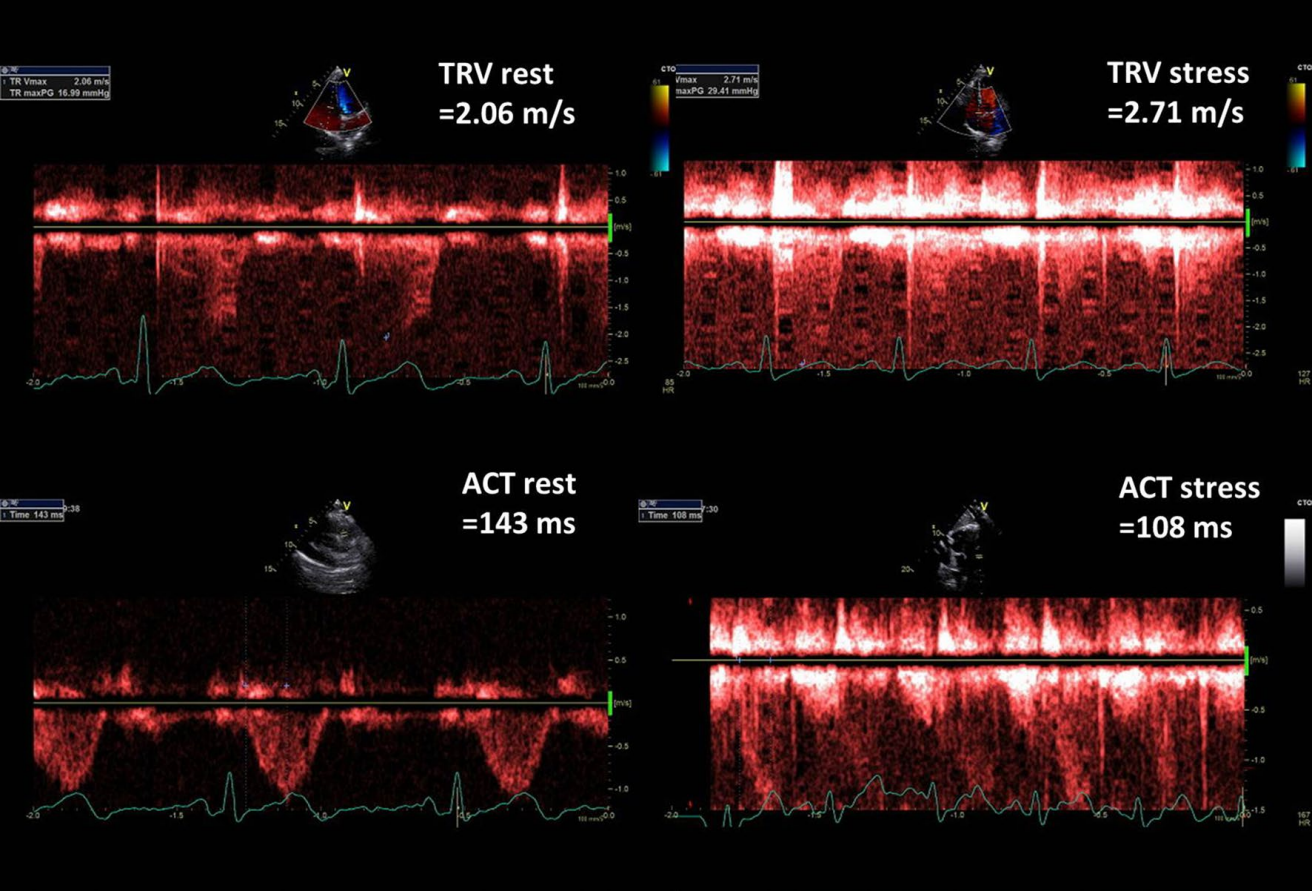
Graphic presentation of TRV and ACT measurements at baseline and peak stress in 43-year-old woman with very good exercise tolerance and high normal PVRI, calculated as the ratio of ESE time and ΔSPAP in tens; PVRI = 14 min/1.3 = 10.8

**Fig. 3:**
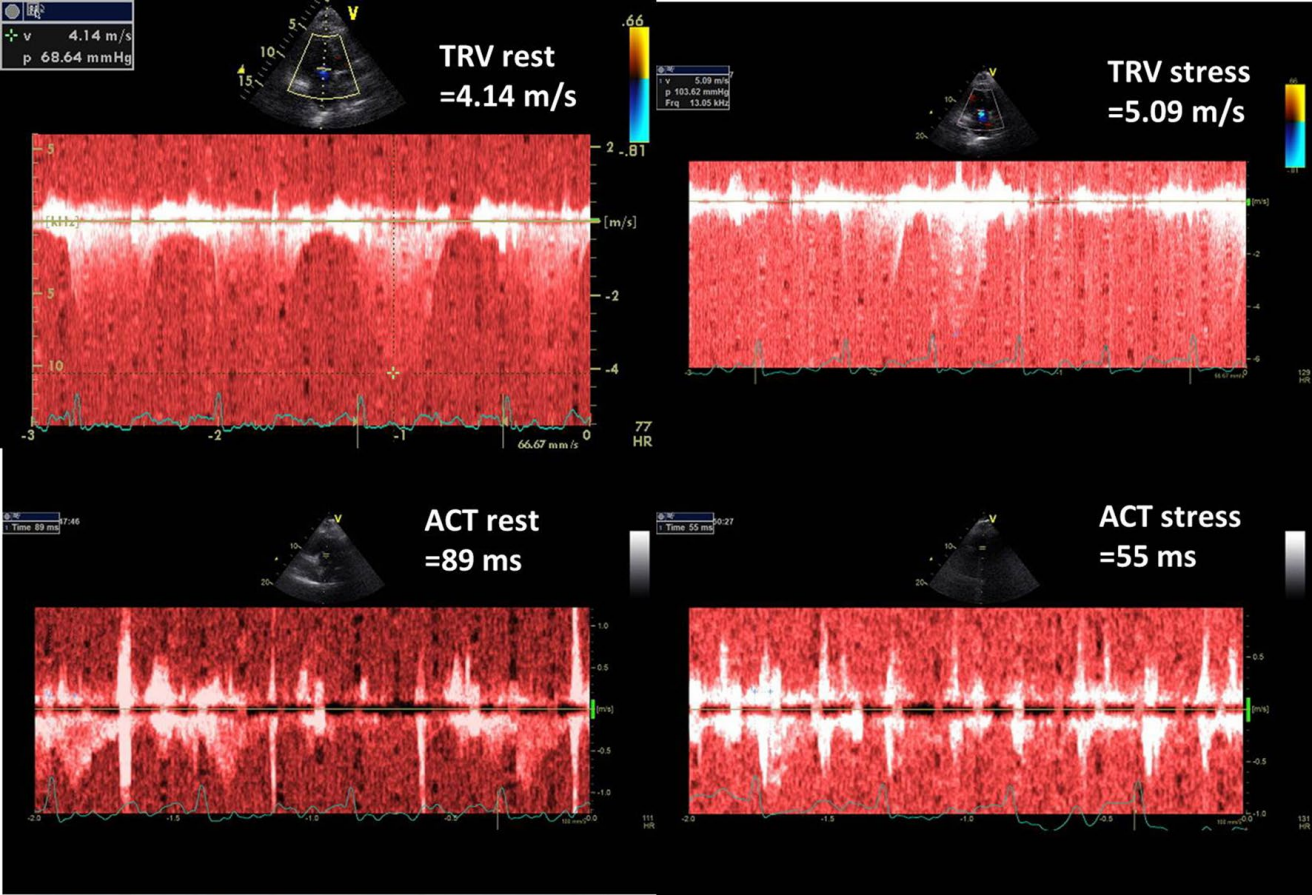
Graphic presentation of TRV and ACT measurements at baseline and peak stress in 63-year-old man with poor exercise tolerance and highly abnormal PVRI, calculated as the ratio of ESE time and ΔSPAP in tens; PVRI = 3 min/3.5 = 0.9

**Fig. 4:**
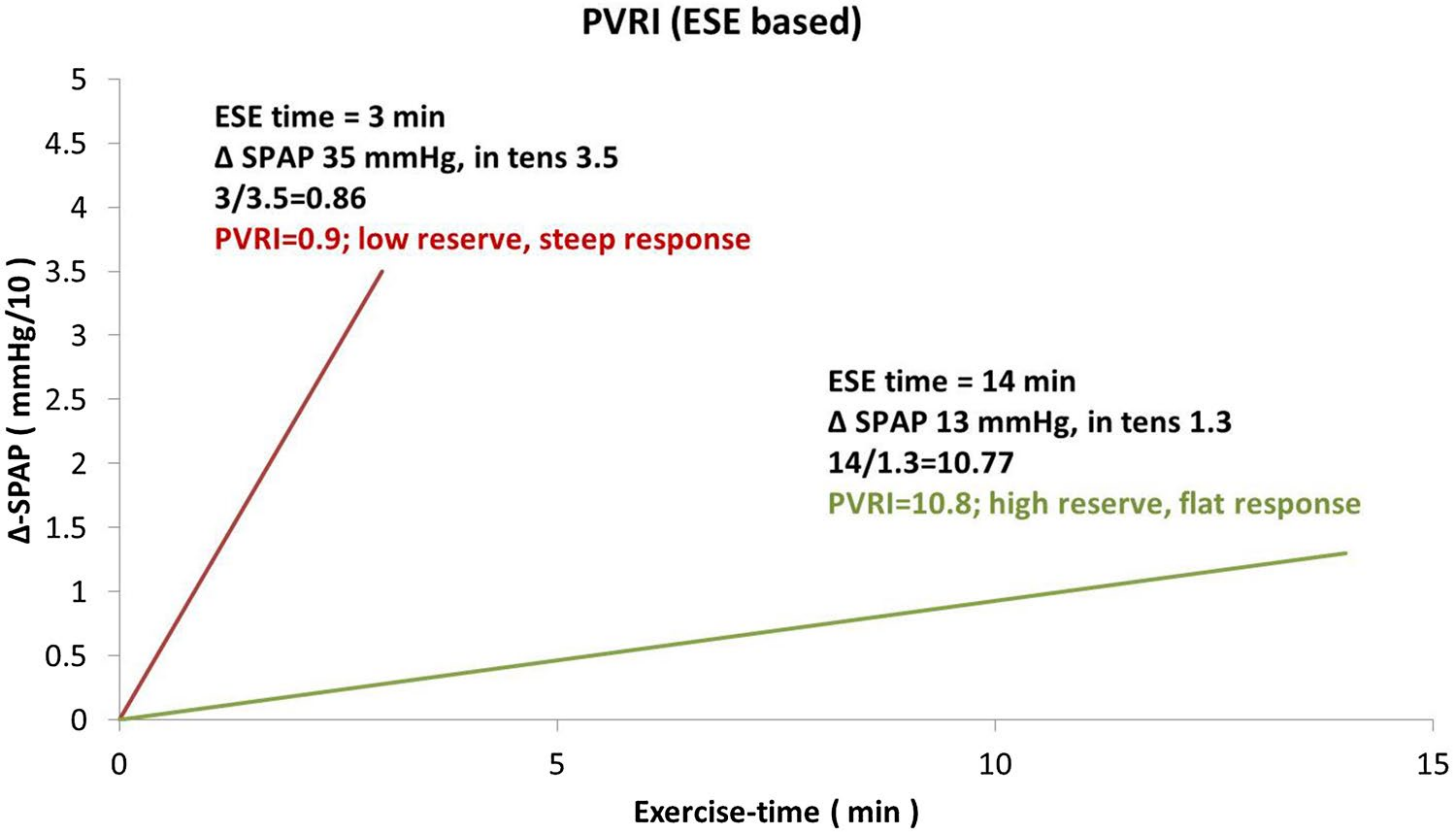
Graphic comparison of PVRI data representive for healthy subject with high value of PVRI and flat increase of SPAP during exercise and abnormal steep time—pressure relationship in patient with PH of diagnosed chronić thromboembolic origin

We added three figures as case-illustrating graphics. Figure 2 presents TRV and ACT rest and stress measurements done in healthy 43-old-year female whereas in Fig. 3 respective parameters are displayed in 63-year-old man with diagnosed pulmonary hypertension. Figure 4 illustrates the comparison of PVRI expressed as slopes of delta SPAP in relations to ESE time. The lines illustrate respectively an normal flat response (with high PVRI) and a steep impaired response, with low PVRI value.

### Statistical analysis

Data are expressed as mean ± standard deviation for continuous or frequency for categorical data. The distribution was assessed with the D'Agostino-Pearson test and adequate tests were used. For correlation Pearson’s or Spearman coefficients were calculated. The comparisons were performed with t-test for independent samples or paired t-test, Wilcoxon test or chi-squared test for categorical data. Statistical significance was set at p < 0.05. ROC analysis was applied for detection of cut-off values for studied parameters. Analyses were conducted with MedCalc V. 12.1.4. (Frank Schoonjans Belgium).

## Results

The clinical characteristics and hemodynamic data related to ESE of the patients and controls are listed in Table 1. In patients with diagnosed PH 11 had idiopathic PAH, 8 congenital heart disease related PH, 6 connective tissue related PH and 6 chronic thromboembolic PH. In PH at risk group, associated disease was systemic sclerosis in 56 patients, and mixed connective tissue disease in five patients.

**Table 1:** Clinical and hemodynamics characteristics of the patients

Variable	Group 1 PH, N = 31	Group 2 PH risk, N = 61	Group 3 controls, N = 50	p 1 vs 2	p 2 vs 3	p 1 vs 3
Age (years)	56 ± 18 (19–87)	57 ± 15 (19–80)	52 ± 14 (21–88)	ns	ns	ns
Sex (males/females, males %)	14/17 (45%)	9/52 (15%)	23/27 (46%)	0.0039	0.0007	ns
BSA (m^2^)	1.84 ± 0.2 (1.44–2.44)	1.75 ± 0.2 (1.4–2.64)	1.91 ± 0.2 (1.43–2.44)	0.044	0.0001	ns
Body mass index (kg/m^2^)	26 ± 4.8 (18.5–36.5)	26.8 ± 4.9 (18.4–37.2)	26.5 ± 4.4 (18.9–39.3)	ns	ns	ns
Hypertension	13 (42%)	30 (49%)	17 (34%)	ns	ns	ns
Diabetes	3 (10%)	4 (5%)	2 (4%)	ns	ns	ns
Smoking	0 (0%)	18 (30%)	8 (16%)	0.0017	ns	0.0497
ACE-I	13 (42%)	13 (21%)	15 (30%)	ns	ns	ns
CCB	5 (16%)	20 (33%)	3 (6%)	ns	0.0012	ns
Diuretics	22 (71%)	22 (36%)	7 (14%)	0.0031	0.016	< 0.0001
HR rest (bpm)	75 ± 14 (48–110)	79 ± 12 (55–120)	68 ± 13 (48–100)	ns	< 0.0001	0.0249
HR peak (bpm)	119 ± 19 (85–173)	130 ± 17 (90–170)	131 ± 22 (78–170)	0.0059	ns	0.0141
DBP rest (mmHg)	80 ± 11 (61–106)	75 ± 10 (50–100)	81 ± 14 (51–115)	0.031	0.0098	ns
DBP peak (mmHg)	91 ± 17 (43–127)	88 ± 15 (69–141)	96 ± 21 (65–181)	ns	0.0213	ns
SBP rest (mmHg)	128 ± 20 (96–169)	123 ± 16 (80–160)	134 ± 19 (97–174)	ns	0.0013	ns
SBP peak (mmHg)	157 ± 30 (99–224)	161 ± 25 (126–230)	182 ± 31 (122–240)	ns	0.0001	0.0006
O_2_ saturation rest	91 ± 8 (70–99)	96 ± 3.4 (88–99)	97 ± 1.7 (93–99)	0.0001	ns	< 0.0001
O_2_ saturation peak	83 ± 13 (55–100)	94 ± 5.6 (78–99)	96 ± 2.9 (81–100)	< 0.0001	0.0242	< 0.0001
Workload (Watt)	70 ± 20 (50–100)	86 ± 24 (45–150)	133 ± 42 (50–200)	0.002	< 0.0001	< 0.0001
Exercise time (min)	5.6 ± 1.6 (4–8)	6.8 ± 2 (4–12)	10.6 ± 3.3 (4–16)	0.0047	< 0.0001	< 0.0001
Tiredness in Borg Scale	7.8 ± 1.3 (4–100)	5.3 ± 1.8 (3–10)	7.6 ± 0.7 (6–9)	< 0.0001	< 0.0001	ns

*ACE-I* inhibitors of angiotensin converting enzyme, *BSA* body surface area, *CCB* calcium channel blockers, *PH* pulmonary arterial hypertension, *DBP* diastolic blood pressure, *SBP* systolic blood pressure, *HR* heart rate, *PH* pulmonary arterial hypertension

The groups did not differ according to mean age, body mass index and the prevalence of hypertension and diabetes, nevertheless in PH risk group higher prevalence of women as well as smaller mean body surface area were observed. Contrary to both PH at risk and control group no patients with confirmed PH smoked.

The achieved workload as well as exercise duration (10.6 ± 3.3 vs 6.4 ± 1.9 vs 5.6 ± 1.6 min) were greater in controls as compared with PH risk and PH and was reflected also in more elevated peak systolic pressure (182 ± 31 vs 161 ± 25 vs 157 ± 30 mmHg).

For the variability assessment of echocardiographic parameters we calculated intraclass correlation coefficient. Intraobserver variability achieved for ACT at rest 0.96 and for TRV 0.99, whereas for the peak parameters these values were 0.89 for ACT and 0.96 for TRV, as we previously reported [9].

**Fig. 5:**
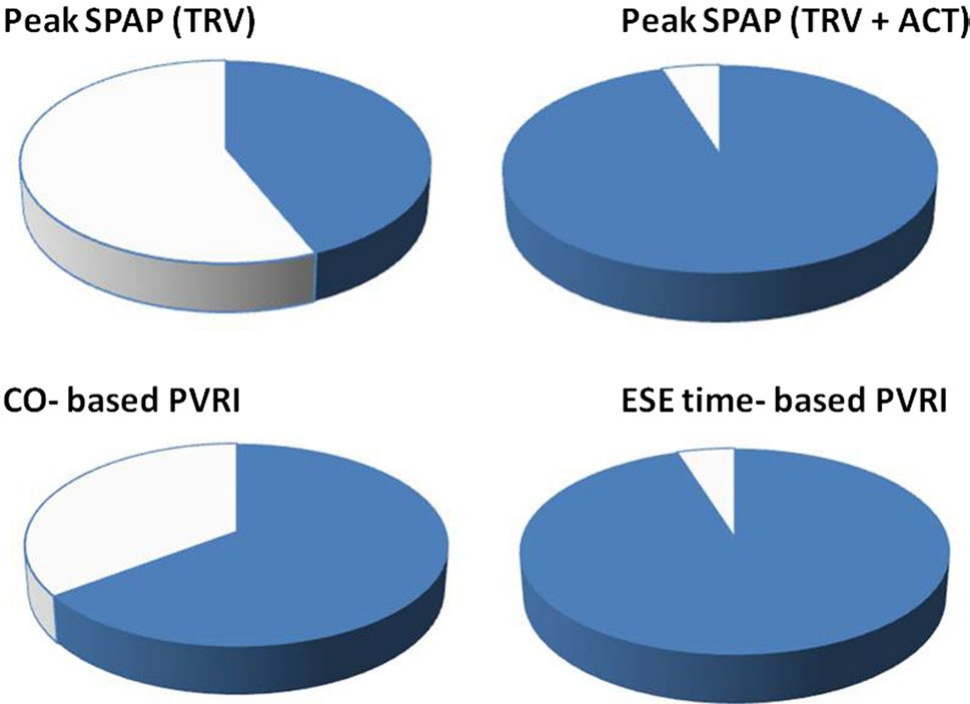
Graphic presentation of the feasibility of echocardiographic parameters in the whole studied group. Including the ACT as an alternative parameters for SPAP estimation increased feasibility of stress SPAP obtaining from 44 to 95%. Similarly, the substitution of CO with ESE time increased feasibility for flow-independent indices from 65 to 95%. *ACT* acceleration time, *CO* cardiac output, *ESE* exercise stress echocardiography, *PVRI* pulmonary vascular reserve index, *SPAP* systolic pulmonary artery pressure, *TRV* tricuspid regurgitant velocity. Blue colour—feasible data, white—unfeasible data; feasibility results: Peak SPAP (TRV based) = 44%, Peak SPAP (TRV + ACT based) = 95% CO based PVRI = 65%, ESE time-based PVRI = 95%

In Table 2 the feasibility of echocardiographic parameters is displayed showing for peak of ESE 44% of TRV feasibility, 77% for ACT and 95% for SPAP estimation based on both TRV and ACT according to TRACT protocol. Similarly, the application of ESE time in the place of CO enabled the significant feasibility improvement of PVRI from 65 to 95%, p < 0.0001, see Fig. 5.

**Table 2:** Echocardiographic findings: feasibility expressed as number and percentage

Parameter	Number in the whole group of 142 pts	(%) in the whole group
Rest TRV	80	56
Rest ACT	117	82
Peak TRV	62	44
Peak ACT	109	77
SPAP (TRV based) rest	80	56
SPAP (TRV based) peak	62	44
SPAP rest (TRV + ACT based)	138	97
SPAP peak (TRV + ACT based)	135	95
PV Resistance rest (Wood U)	79	56
PV Resistance peak (Wood U)	60	42
CO-based PVRI	92	65
ESE time- based PVRI	135	95

*CO* cardiac output, *ESE* exercise stress echocardiography, *PV resistance* pulmonary vascular resistance, calculated according to Abbas formula: PV Resistance (Wood Units) = TRV (m/s)/VTI RVOT (cm) *10 + 0.16. *PVRI* pulmonary vascular reserve index, *TRV* tricuspid regurgitant velocity, *ACT* acceleration time of pulmonary flow, *SPAP* systolic pulmonary artery pressure

In Table 3 the comparison of echocardiographic parameters in all three groups showed the consistent differences for parameters reflecting pulmonary pressure, resistance and reserve indicating the most advanced impairment of pulmonary circulation status in PH group, intermediate stage in PH risk patients and normal or near-normal values in age-matched controls. Additionally we estimated also the both PVRI parameters in 29 healthy, young patients (mean age 32 ± 5.3 years, 21 males, cardiovascular risk factors including smoking and obesity excluded) obtaining reference, normal values of 5.26 ± 2.9 L/min/mmHg for CO-based PVRI and 5.03 ± 2.59 (min/mmHg) for ESE time-based PVRI.

**Table 3:** Echocardiographic data comparison

Parameter	Group 1 PH	Group 2 PH risk	Group 3 controls	p 1 vs 2	p 2 vs 3	s 1 vs 3
Rest TRV (cm/s)	390 ± 102 (150–567)	244 + 63 (120–380)	219 ± 51 (133–390)	< 0.0001	ns	< 0.0001
Rest ACT (ms)	68 ± 18 (42–129)	112 ± 32 (54–177)	112 ± 23 (65–175)	< 0.0001	ns	< 0.0001
Stress TRV (cm/s)	480 ± 82 (320–620)	353 ± 71 (260–535)	254 ± 70 (148–330)	< 0.0001	0.0001	< 0.0001
Stress ACT (ms)	59 ± 18 (28–111)	84 ± 20 (41–120)	95 ± 21 (65–152)	< 0.0001	0.0238	< 0.0001
SPAP (TRV based) rest	72 ± 33 (12–143)	32 ± 11 (11–58)	24 ± 11 (12–69)	< 0.0001	0.0137	< 0.0001
SPAP (TRV based) stress	110 ± 32 (56–169)	55 ± 24 (29–129)	33 ± 14 (15–50)	< 0.0001	0.0021	< 0.0001
SPAP rest (mmHg)	70 ± 33 (12–143)	26 ± 10 (10–58)	23 ± 10 (12–69)	< 0.0001	ns	< 0.0001
SPAP stress (mmHg)	101 ± 36 (29–169)	45 ± 19 (10–129)	32 ± 12 (15–59)	< 0.0001	0.0001	< 0.0001
PVR rest (Wood U)	3.9 ± 2.0 (1.2–9.0)	1.62 ± 0.51 (0.73–2.7)	1.7 ± 0.7 (0.9–4.6)	< 0.0001	ns	< 0.0001
PVR stress (Wood U)	4.6 ± 2.0 (2.1–9.0)	2.0 ± 0.83 (1.2–5.0)	1.7 ± 0.9 (0.8–4.7)	< 0.0001	ns	< 0.0001
CO-based PVRI (L/min/mmHg)	1.0 ± 0.95 (0.23–5.4)	1.57 ± 0.47 (0.74 ± 2.7)	4.28 ± 2.3 (0.49–10.8)	0.034	< 0.0001	< 0.0001
ESE time-based PVRI (min/mmHg)	0.66 ± 0.39 (0.27–2.1)	1.89 ± 1.47 (0.31–10)	3.95 ± 2.26 (0.8–11)	< 0.0001	< 0.0001	< 0.0001

*ACT* acceleration time of pulmonary flow, *CO* cardiac output, *ESE* exercise stress echocardiography, *N'* number of feasible measurements, *PV resistance* pulmonary vascular resistance, *PVRI* pulmonary vascular reserve index, *SPAP* systolic pulmonary artery pressure, *TRV* tricuspid regurgitant velocity

**Fig. 6:**
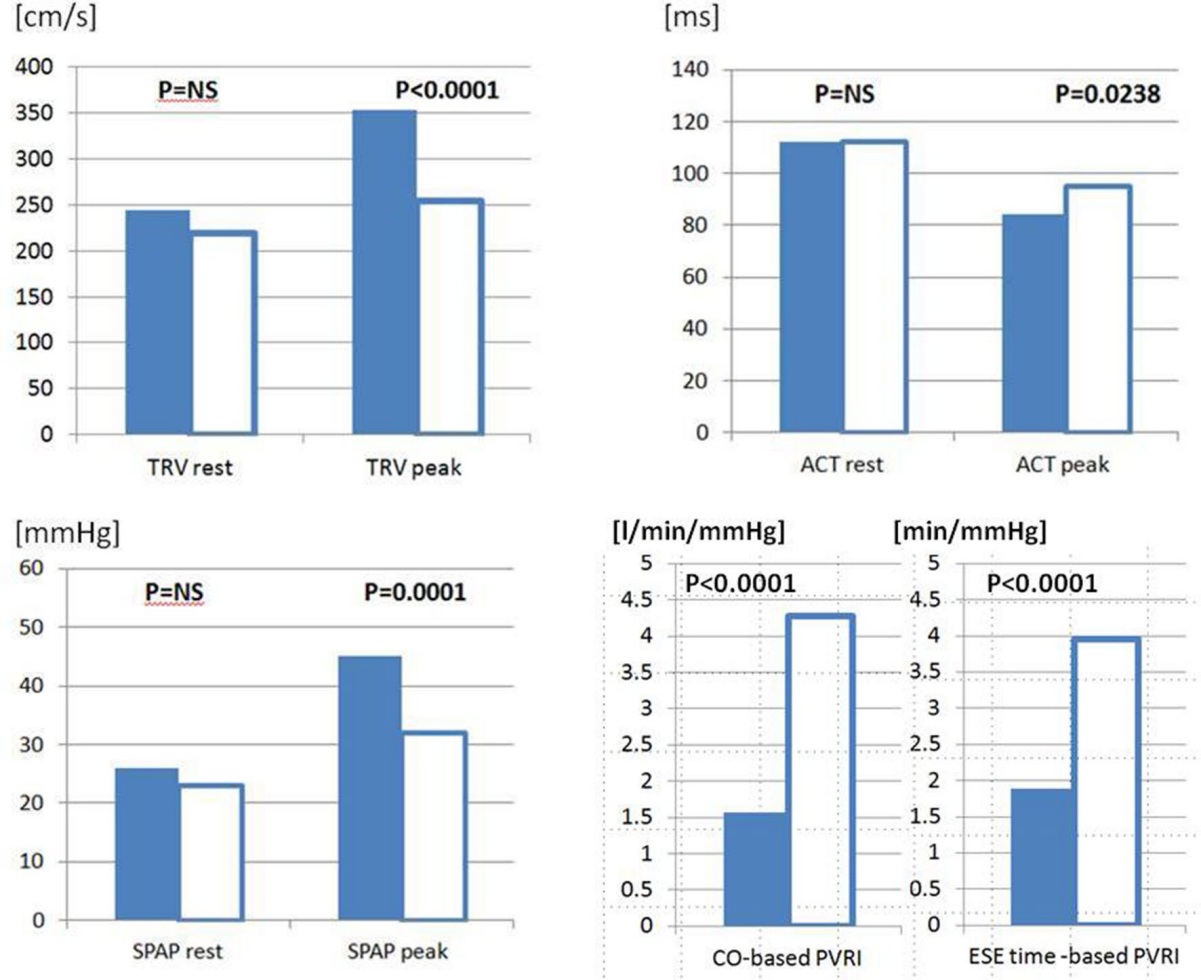
The subgroup analysis- comparison between patients at risk of PH and controls. Bar graph showing the separation of the subgroup PH risk and controls, empty columns only by indices measured at peak ESE and pulmonary vascular reserve index. From left to right: TRV rest; TRV peak, (cm/s) ACT; rest, ACT peak (ms), SPAP rest, SPAP peak (mmHg), CO-based PVRI (CO/SPAP*10^–1^) (l/min/mmHg); ESE time-based PVRI (ESE time/SPAP*10^–1^) (min/mmHg). *ACT* acceleration time, *CO* cardiac output, *ESE* exercise stress echocardiography, *PVRI* pulmonary vascular reserve index, *SPAP* systolic pulmonary artery pressure, *TRV* tricuspid regurgitant velocity

Moreover, in the analysis focused on the comparison between patients at risk of PH and controls (group 2 and 3) all resting parameters: TRV, ACT, SPAP and PVR were similar in both compared groups, whereas variables measured at peak stage of ESE (apart from pulmonary vascular resistance, PVR) revealed higher values of TRV and SPAP and shorter ACT in patient with risk factors for PH, as well as significantly higher PVRI (either CO-based or exercise-time-based) in controls, see Fig. 6.

Having the subgroup of invasively proved diagnosis of PH (31 patients) we estimated from ROC analysis cut-off values for flow dependent and independent parameters. The results are displayed in Table 4. Estimated cut-off values were tested in group at risk of PH development. The best cut-off values were: > 62 mmHg for SPAP, TRV-based; > 69 mmHg for SPAP, TRACT-based; ≤ 1.29 for CO-based PVRI; and ≤ 1 for ESE time- based PVRI.

**Table 4:** Diagnostic value and cut- offs for flow dependent and independent parameters in the diagnosis of confirmed PH, the results of the ROC analysis

Parameter	AUC	Cut-off value	Sensitivity	Specificity
SPAP (TRV based) stress (mmHg)	0.964	> 62	96%	89%
SPAP (TRV + ACT based) stress (mmHg)	0.935	> 69	87%	98%
CO-based PVRI (L/min/mmHg)	0.922	≤ 1.29	84%	95%
ESE time-based PVRI (min/mmHg)	0.949	≤ 1.0	87%	91%

*ACT* acceleration time of pulmonary flow, *AUC* area under curve, *CO* cardiac output, *ESE* exercise stress echocardiography, *PVRI* pulmonary vascular reserve index, *SPAP* systolic pulmonary artery pressure, *TRV* tricuspid regurgitant velocity

Moreover, we tried to estimate also the cut offs for diagnosis PH risk patients among subgroup consisted of PH at risk and age –matched controls and found the values ≤ 1.9 for CO-based PVRI; and ≤ 1.7 for ESE time- based PVRI with respective ROC AUC of 0.917 and 0.824.

**Fig. 7:**
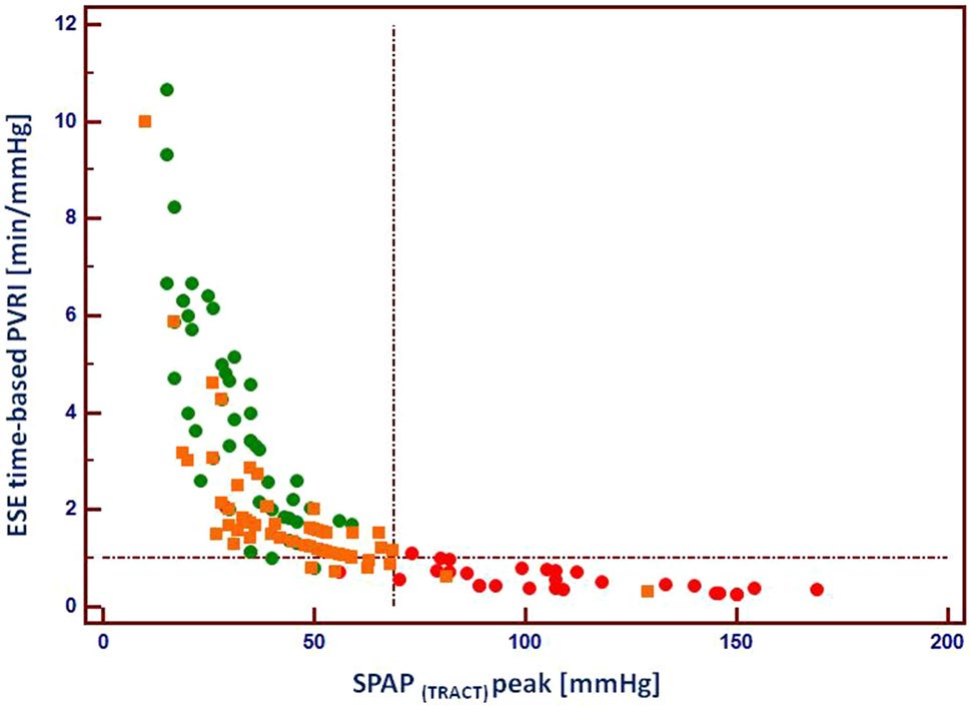
The relationship between ESE-time based pulmonary vascular reserve index and SPAP achieved at peak ESE, showing clear separation of controls (green circles) and patients with confirmed PH (red circles). As far as group at risk of PH is concerned (orange squares) two patients showed SPAP at peak > 69 mmHg whereas 11 patients displayed lowered pulmonary vascular reserve < 1 min/mmHg, which suggests higher sensitivity of this parameter for detection of early stage of pulmonary hemodynamics impairment. *ESE* exercise stress echocardiography, *PVRI* pulmonary vascular reserve index, *SPAP* systolic pulmonary artery pressure, *TRACT* acronym for using TRV and ACT for SPAP estimation, vertical line indicates cut-off for SPAP 69 mmHg, horizontal line cut-off value for ESE time-based PRVI 1 min/mmHg, green circles—controls, orange squares—patients at risk of PH, red circles—patients with confirmed PH

The relationship between peak SPAP calculated according to TRACT approach and ESE-time based PVRI is displayed in Fig. 7 illustrating non-linear relationship between both parameters and high discriminatory value for three examined subgroups.

## Discussion

Noninvasive estimates of PVRI expressed as indices based on CO or exercise-time can be obtained during exercise with moderate (CO-based) or excellent (ESE-time based) success rate, achieving 95% feasibility for simplified data when avoiding CO calculation and utilizing beside TRV also ACT for SPAP calculation, Fig. 5. These PVRI parameters allow a clear discrimination of patients with PH or at risk for PH development as compared to control subjects as shown in Figs. 6 and 7. The significance of the pulmonary hemodynamic assessment at peak stress was especially pronounced when applied for detection of patients at risk of PH, revealing similar values as controls at rest, see Fig. 6. The application of indexed to flow indices seems to be more specific for accurate comparison of pulmonary vascular function between patients achieving highly varied level of exercise and related with this different peak cardiac output. In our study we observed significant percentage of patients achieving peak SPAP values between 40 and 60 mmHg for which more flow independent indices seem to offer important additional discriminating value, see Fig. 7. The flow correction can be obtained with Doppler-based measurement of stroke volume and CO (which is the recommended method for the estimation of CO in echocardiography) or with the means of volumetric only 2D assessment of the left ventricle, but without detectable loss of information also with achieved workload or simpler exercise-time, an imaging independent proxy of cardiac output.

### Comparison with previous studies

Several studies have shown the value of ACT for evaluation of pulmonary pressures (mean PA or SPAP) when TRV is not available, which happens frequently especially at peak exercise [8, 16, 28]. Our data are also consistent with previous evidences showing that pulmonary vascular resistance and reserve may be more informative than SPAP in identifying initial alterations of pulmonary hemodynamics [29, 30]. Simplified relationship between SPAP and exercise intensity have similar accuracy compared to direct measurements of CO in identifying an abnormal pulmonary vascular reserve, as shown by Claessens et al. in patients undergoing a simultaneous invasive assessment of pulmonary pressures [11]. In their study, an abnormal pulmonary reserve was identified as mPAP/CO slope by exercise CMR with invasive assessment of pressures. This method has superb accuracy but limited appeal in the clinical arena due to invasiveness and cost.

Yet, the noninvasive parameters which we proposed offered simple and highly accurate cut-offs for identification of PH patients with CO-based PVRI ≤ 1.29 and time-based PVRI ≤ 1 reflecting in an intuitive manner severely limited PVR, whereas the respective values ≤ 1.9 and ≤ 1.7 may indicate on early stage of pulmonary hypertension in which resting parameters are still near-normal. The availability of a highly feasible and simplified index is the prerequisite for large scale multicenter testing in the clinical arena [12, 13, 20].

### Clinical implications

Pulmonary hemodynamics is of utmost importance in the diagnosis, risk stratification and treatment in many cardiovascular conditions, from heart failure to pulmonary hypertension, extreme physiology and congenital heart disease [31, 32] with Doppler echocardiography being the most widely used screening tool in current clinical practice [14].

Yet, the noninvasive assessment of pulmonary hemodynamics with ESE does not find a relevant place in evidence-based recommendations, in spite of the recognized conceptual merits of evaluating hemodynamics during stress which can unmask early disease [33]. European Society of Cardiology 2015 guidelines on pulmonary hypertension clearly state that "exercise echo has technical and methodological limitations and is NOT recommended for PH/PAH screening" (class of evidence III, level of recommendation C) [2]. More recently, the 2019 European Society of Cardiology consensus recommendations on heart failure with preserved ejection fraction propose ESE with diastolic stress test in patients with intermediate probability on the basis of resting functional, morphologic and biochemical criteria, but also specify that peak TRV is only feasible in 50% of patients, cannot be used as a stand-alone criterion due to its flow-dependence, and a peak TRV > 3.4 m/s has some diagnostic value only when accompanied by E/e′ > 15 during exercise stress [34]. At least in principle, exercise-time based PVRI approach might remove the feasibility and conceptual barriers currently limiting an extensive ESE use in the clinical arena.

**Fig. 8:**
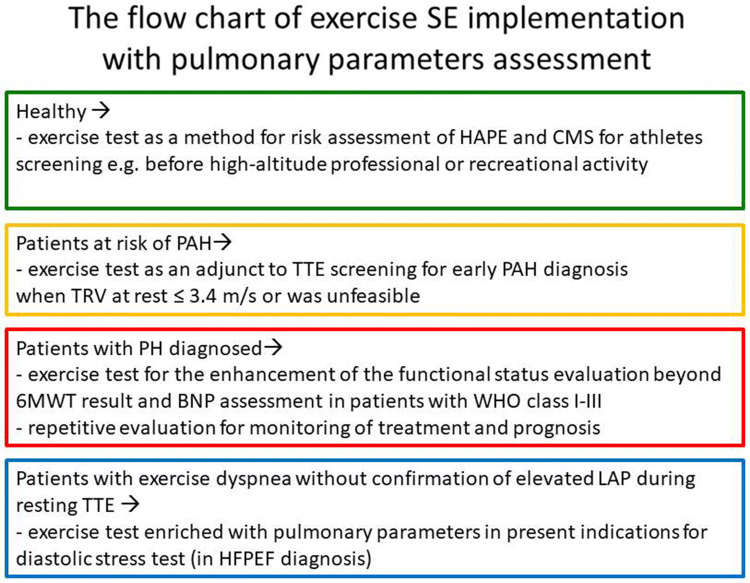
Proposed role of the exercise stress in PH diagnosis. *BNP* brain natriuretic peptide, *CMS* chronic mountain sickness, *HAPE* high altitude pulmonary edema, *HFPEF* heart failure with preserved ejection fraction, *PAH* pulmonary arterial hypertension, *PH* pulmonary hypertension, *TRV* tricuspid regurgitant velocity, *TTE* transthoracic echocardiography, *6MWT* six minute walk test

Despite its present limitations and with accordance to recent guidelines [2, 14] exercise stress echocardiography with noninvasive assessment of pulmonary pressure, resistance and right ventricular function should be considered in patients at high risk for PAH development as the adjunct tool exceeding the diagnostic potential of rest-only examination. Similarly, in patients with the established diagnosis of PH, not showing the contraindications to exercise, noninvasive but quantitative assessment could improve monitoring of treatment and help further in prognostic stratification. Moreover, there are data showing that ESE, beyond the more technically demanding hypoxic challenge, may predict the risk of chronic mountain sickness (CMS) and high altitude pulmonary edema (HAPE) appearance [35]. Additionally, the adding the pulmonary parameters to the assessment of so called „diastolic stress echo” have a potential for more detailed stratification of these the most prevalent etiology of „secondary” PH [10], see Fig. 8 displaying the proposed role of the exercise stress in PH diagnosis.

### Limitations

Groups were not homogeneous, in particular with higher prevalence of women in the group of PH risk, due to the well known epidemiological profile of connective tissue disease. However, the pulmonary pressure response during exercise seems to be similar between men and women as has been recently published in metaanalysis of studies basing on right heart catheterisation [36]. Other possible confounders are intergroup inhomogeneities in BSA, smoking and cardiovascular drug usage, unavoidable when comparing group with different female to male ratio as well as different health status. However, the primary aim of the study concerning the feasibility assessment of the new proposed method, was unlikely affected by these confounders.

TRV and ACT have recognized limitations in the assessment of SPAP, including inadequate validation during exercise, need to include during exercise right atrial pressure typically assumed from inferior vena cava dimension and collapsibility at rest, possible heart rate-dependence for ACT.

The echocardiographers were not blinded to the study condition since the increased heart rate is associated with peak exercise. Also the acquisition of both parameters was not simultaneous but data were recorded in sequential manner.

Finally, we did not use saline microbubble injection as an attempt to enhance TRV spectrum since this maneuver requires intravenous injections and further complication of an already technically demanding ESE study.

## Conclusion

Patients at risk for PH can be effectively separated from controls using transthoracic echocardiographic assessment during ESE. To achieve this clinically important goal, flow-independent indices such as CO-based PVRI or exercise time-based PVRI may be more effective than resting or stress indices such as SPAP. The feasibility of assessing PVRI is excellent if SPAP is obtained combining TRV or ACT, whenever TRV is not feasible, along with imaging-independent exercise-time used as a simple proxy of CO. This integrated approach allows to increase the success rate close to 100% and to adopt a more robust and more flow-independent assessment of pulmonary hemodynamics into practice.

## Abbreviations

ACT: Acceleration time of physiologic systolic forward pulmonary flow velocity
CO: Cardiac output
CO/SPAP: CO based pulmonary vascular reserve index
ESE: Exercise stress echocardiography
ESE time/SPAP: ESE time based pulmonary vascular reserve index
PAH: Pulmonary arterial hypertension
PH: Pulmonary hypertension
PVRI: Pulmonary vascular reserve index
RAP: Right atrial pressure
SPAP: Systolic pulmonary artery pressure
TRACT: Acronym for using TRV and ACT for SPAP estimation
TRV: Tricuspid regurgitant velocity
TTE: Transthoracic echocardiography
